# Effect and Mechanism of Si-Miao-Yong-An on Vasa Vasorum Remodeling in ApoE^−/−^ Mice with Atherosclerosis Vulnerable Plague

**DOI:** 10.3389/fphar.2021.634611

**Published:** 2021-04-14

**Authors:** Meng Li, Zhongwen Qi, Junping Zhang, Ke Zhu, Yueyao Wang

**Affiliations:** ^1^ First Teaching Hospital of Tianjin University of Traditional Chinese Medicine, Tianjin, China; ^2^ Tianjin University of Traditional Chinese Medicine, Tianjin, China

**Keywords:** Si-Miao-Yong-An, vasa vasorum, atherosclerosis, vulnerable plague, ApoE^–/–^ mice

## Abstract

**Objective:** To observe the effect of Si-Miao-Yong-An (SMYA) on atherosclerosis (AS) vulnerable plaques, and to further explore the mechanism by vasa vasorum (VV) angiogenesis and maturation as an entry point.

**Methods:** SPF-class healthy male ApoE^−/−^ mice were randomized into model group, simvastatin group and SMYA group, and C57BL/6 mice were used as the control group. After 8 weeks of intervention, the pathological morphology of plaque was observed by HE staining; the VV density in plaque and aortic adventitia were observed by immunohistochemistry; VV maturation was measured by double-labelling immunofluorescence; the critical proteins of HIF-1α-Apelin/APJ and Ang-1/Tie signal pathways were detected by western blotting.

**Results:** SMYA decreased the plaque area and the ratio of plaque to lumen area; increased the minimum thickness of fibrous cap and its effect was greater than simvastatin. SMYA suppressed the VV neovascularization; promoted smooth muscle cells recruitment and VV maturation, which maintained plaque stability; its effect was obviously superior to simvastatin. SMYA deceased the expression of HIF-1α, Apelin, APJ, Phospho-MEK1/2 (Ser217/221), Phospho-p44/42 MAPK (Erk1/2) (Thr202/Tyr204), Phospho-p70 S6 Kinase (Thr421/Ser424), Ang-2 and Tie-2; it also increased the expression of Ang-1, Phospho-Akt (Ser473), Phospho-FOXO1 (Ser256) and Survivin.

**Conclusions:** SMYA can decrease the AS plaque area in ApoE^−/−^ mice, suppress the VV neovascularization and promote the VV maturation, and stabilize AS vulnerable plaque. The mechanism could be regulating the HIF-1α-Apelin/APJ and Ang-1/Tie signal pathways.

## Introduction

In recent years, the research on AS has gradually changed from the “inside-out”—inflammatory response mechanism focusing on the endangium to “outside-in” (Ruan et al., 2013; Fatkhullina et al., 2016). There are abundant VV networks in adventitia and the outer third of the vascular media of large and medium-sized artery, which delivers oxygen and nutrients to the host vessel wall and discharges metabolic waste, maintains the material metabolism and energy balance of the host vessel, and keeps the structure and function integrity of the host vessel (Majesky, 2015). A growing number of researches show that VV neovascularization is essential to AS (Boyle et al., 2017). Metal cannulae-tied transparent visualization of human coronary arteries established an association of VV in AS progression and associated sequelae (Barger et al., 1984). Another study observed the coronary arteries of hyperlipidemia pigs and found abnormal hyperplasia and increased density of adventitia VV at the early stage of AS, and the VV density was positively correlated with the degree of AS lesions (Gössl et al., 2003). The pathologic neovascularization has structural defects, with great brittleness, high leakage and being easy to rupture and bleed. Instability and vulnerability of plaques cause the major clinical risks of AS lesions (Xu et al., 2015). VV neovascularization in plaques is a critical factor to promote the stable plaques changing into vulnerable plaques, which is closely related to the occurrence of intra-plaque hemorrhage, plaque rupture and clinical cardiovascular and cerebrovascular events (Li et al., 2017).

SMYA, a Chinese herbal formulation comprising Flos Lonicerae Japonicae, Radix Scrophulariae Ningpoensis, Radix Angelicae Sinensis and Radix Glycyrrhizae Uralensis, has been widely used in treating AS diseases, such as coronary heart disease, lower extremity atherosclerotic occlusive disease, cerebral infarction, etc. Clinical and experimental studies have shown that SMYA can inhibit the inflammatory response and antagonize the blood clotting process (Liu et al., 2019; Zheng et al., 2019). However, can SMYA improve the AS plaque stability in ApoE^−/−^ mice? In the current study, we investigated the effect of SMYA on VV remodeling in ApoE^−/−^ mice with AS vulnerable plague and explored its mechanism of action.

## Materials and Methods

### Animals

Male ApoE^−/−^ mice aged 7–8 weeks, weight (23.46 ± 1.36) g were purchased from Beijing Huafukang Bioscience Co., Ltd (certificate No. SCXK Beijing 2014-0004). C57BL/6 mice aged 7–8 weeks, weight (22.67 ± 1.29) g were purchased from Beijing Weitonglihua experimental animal technology Co., Ltd (certificate No. SCXK Beijing 2012-0001). The protocol was approved by the Animal Ethics Committee of Tianjin University of Traditional Chinese Medicine (No. TCM-LAEC2018032), China.

### Materials

SMYA (Lyophilized powder composed of *Scrophularia ningpoensis Hemsl., Lonicera japonica., Angelica Sinensis., Glycyrrhiza uralensis Fisch*, prepared according to the ratio 3:3:2:1 and purchased from the first affiliated hospital of Tianjin university of Traditional Chinese Medicine; Simvastatin Tablets (Hangzhou MSD pharmaceutical Co., Ltd, Lot number: J20130068); l-methionine (Sigma); Saturated oil red O stain (Beijing Solarbio Technology Co., Ltd.); Hematoxylin-eosin stain kit (Tianjin Baihao Biotechnology Co., Ltd.); Rabbit monoclonal anti-CD34 (Abcam); Rabbit polyclonal anti-HIF-1 Alpha (Beijing Bioss Biotechnology Co., Ltd); Rabbit polyclonal anti-Apelin-13 (Beijing Bioss Biotechnology Co., Ltd); Rabbit polyclonal anti-Apelin Receptor (Beijing Bioss Biotechnology Co., Ltd); Rabbit polyclonal anti-phospho-MEK1/2 (Ser217/221) (Cell Signaling Technology); Rabbit monoclonal anti-MEK1/2 (Abcam); Rabbit monoclonal anti-phospho-ERK1/2 (Thr202/Tyr204) (Cell Signaling Technology); Mouse monoclonal anti-ERK1/2 (Beijing Bioss Biotechnology Co., Ltd); Rabbit polyclonal anti-phospho-p70 S6 Kinase (Thr421/Ser424) (Cell Signaling Technology); Rabbit Polyclonal anti-p70 S6 Kinase (Beijing Bioss Biotechnology Co., Ltd); Rabbit polyclonal anti-Ang-1 (Cloud-Clone Corp); Rabbit polyclonal anti-Ang-2 (Cloud-Clone Corp); Rabbit polyclonal anti-Tie-2 (Cloud-Clone Corp); Rabbit monoclonal anti-phospho-Akt (Ser473) (Cell Signaling Technology); Rabbit monoclonal anti-Akt (Beijing Bioss Biotechnology Co., Ltd); Rabbit monoclonal anti-phospho-FoxO1 (Ser256) (Cell Signaling Technology); Rabbit polyclonal anti-FOXO1 (Beijing Bioss Biotechnology Co., Ltd); Rabbit polyclonal anti-Survivin (Cloud-Clone Corp); Mouse monoclonal anti-GAPDH (Abcam).

### Animal Groups and Treatment

The male ApoE^−/−^ mice were randomized into model group, simvastatin group and SMYA group, and C57BL/6 mice were as control group. The ApoE^−/−^ mice were fed a high-fat diet (21% fat, 0.15% cholesterol) with 1.1% l-methionine for 16 weeks. The C57BL/6 mice were fed with normal diet for 16 weeks. After 8 weeks of feeding, the simvastatin group were administered simvastatin with 2.6 mg/kg/d by gavage for 8 weeks; the SMYA group were administered SMYA with 11.7 mg/kg/d by gavage for 8 weeks; the control and model group were given distilled water with 20 ml/kg/d by gavage of the same volume for 8 weeks; Dosage of simvastatin and SMYA were determined by the published pharmacological experiment methodology (Xu, 2002).

### Specimen Collection and Processing

Blood and tissue specimens were harvested at 16 weeks. Each mouse was anesthetized with diethyl ether inhalation. Thoracic and abdominal cavity was quickly opened to expose the heart. PBS buffer was injected into the apex cordis. The aortic root with part of the myocardial tissue were taken and fixed in 4% neutral formaldehyde fixative solution. Aortic tissue was taken and weighed, and was immediately placed in liquid nitrogen for cryopreservation. The aorta tissue specimens were stored to −80°C refrigerator for western blotting.

### Measurement of Atherosclerotic Lesion Area by Oil Red O and HE Staining

Oil Red O staining of aorta: complete aorta was isolated and fixed in 10% neutral formalin for 24 h. The complete aorta was in 60% isopropanol for 10min, stained in oil red O working fluid (3:2) for 3h, washed in 60% isopropanol for 6 times till the background color became white, and then photographed. Image analysis software (Image-Pro plus Version 6.0) was used to analyze and measure the aortic plaque area.

HE staining of aortic root: aortic root was fixed with 10% neutral formalin solution for 24 h. Dewaxing with xylene, conducting gradient alcohol dehydration (100, 95, 75%), conventional paraffin embedding, and continuous coronal sections (5-μm thick) were prepared with gradient alcohol dehydration. Aortic root sections were stained with HE according to HE staining method. Image analysis software (Image-Pro plus Version 6.0) was used to measure the plaque area of aortic root, lumen area, minimum fiber cap thickness and media area, and calculate the plaque area-lumen area ratio.

### VV Density Analysis by Immunohistochemical Staining

The sections were placed in 3% H_2_O_2_ for 10 min, blocked with 5% BSA, and incubated overnight at 4°C with primary antibodies CD34 (1:2500; ab81289, Abcam). The sections were then incubated with secondary antibody for 30 min and visualized using a diaminobenzidine kit. The nucleus was counterstained with hematoxylin (blue). In preliminary experiments, we tested the cross-reactivity between antibodies and rabbit antigens or nonimmune IgG, which was used in negative-control experiments.

Vasa vasorum density in aortic root plaque and aortic adventitia were quantified by counting the total number of CD34-positive microvessels in 5 microscopic fields chosen randomly at ×400 magnification under the microscope. Image analysis software (Image-Pro Plus 6.0) was used to calculate the ratio of positive microvessels to area of aortic root plaque or aortic adventitia (Tonar Jr et al., 2012).

### VV Maturation Analysis by Double Labeling Immunofluorescence

The prepared paraffin sections were dewaxed and processed by 3% H_2_O_2_ for 10 min. Then repair antigen was performed. Sections were blocked with 5% BSA and incubated overnight with primary antibodies against the following: CD34 (1:200; Abcam) and α-SMA (1:100; Abcam). The 2 s antibodies were mixed together, prepared according to 1:1,000 and then incubated overnight at 4°C. DAPI stained for 5 min. The sections were sealed with anti-fluorescence attenuation sealant.

CD34 was the marker of VV and α-SMA was the marker of smooth muscle cell. Image analysis software (Image-Pro Plus 6.0) was used to evaluate the VV maturation. Under the fluorescence microscope, 5 microscopic fields were chosen randomly in plaque at ×400 magnification and calculated the plaque area (PA, mm^2^). Counted the number (N) of mature VV covered by smooth muscle cell. Calculated the density of mature VV in plaque (N/PA) and the average value was taken.

### Measurement of the Critical Proteins of HIF-1α-Apelin/APJ and Ang-1/Tie Signal Pathways by Western Blotting

Aortic tissue proteins were extracted with protein extraction kit. Protein concentration in the supernatants of tissue lysates was measured by BCA Protein Assay Kit. The proteins and a molecular weight marker were separated by 10% SDS–PAGE and transferred to a polyvinylidene difluoride (PVDF) membrane. The membrane was then incubated with primary antibodies for HIF-1 Alpha (1:1,000), Apelin-13 (1:800), Apelin Receptor (1:1,000), phospho-MEK1/2 (Ser217/221) (1:1,000), MEK1/2 (1:5,000), phospho- ERK1/2 (Thr202/Tyr204) (1:1,000), ERK1/2 (1:500), phospho-p70 S6 Kinase (Thr421/Ser424) (1:500), p70 S6 Kinase (1:500), ANGPT1 (1:800), ANGPT2 (1:800), Tie2 (1:500), phospho-Akt (Ser473) (1:1,000), Akt (1:500), phospho-FoxO1 (Ser256) (1:800), FOXO1 (1:500), and Survivin (1:1,000). The antibodies were incubated overnight and reacted with HRP-conjugated secondary antibodies. Extensive washes in 0.05% Tween-20 in TBS were followed by incubation with anti-GAPDH (1:1,000). The blots were then incubated with the chemofluorescent reagent enhanced chemiluminescence (ECL; Thermo Scientific) and exposed to X-ray film in the dark. The intensity of the GAPDH signal was used as an endogenous control, and the band optical density was quantified using Image J (National Institutes of Health, Bethesda, MD).

### Statistical Analysis

All parameters were expressed as mean ± S.D. Statistical analysis was performed using one-way ANOVA followed by the least significant difference (LSD) test for multiple comparisons. SPSS statistical software (version 11.5, SPSS Inc., Chicago, IL, USA) was used for all statistical analyses. The level of significance was set at *p* < 0.05.

## Results

### Effect of SMYA on Atherosclerotic Lesion Area

**FIGURE 1:**
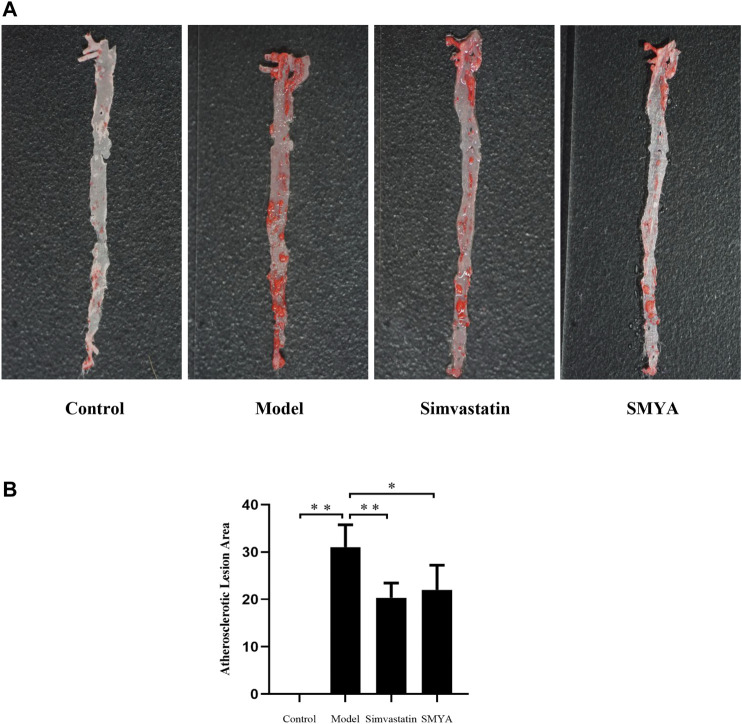
Effect of SMYA on Atherosclerotic Lesion Area. **(A)** oil red o staining showed: in control group, the aorta was milky white, and the aorta intima was smooth and without lipid deposition. Compared with the control group, large areas of continuous flaky red atheromatous plaques could be seen, which was mainly concentrated in the aortic arch and the abdominal aorta and the plaque area of aorta intima was significantly increased in model group. Compared with the model group, only scattered red atherosclerotic plaques could be seen and the plaque area of aorta intima was decreased in both of simvastatin group and SMYA group. **(B)** quantitative analysis of SMYA on atherosclerotic lesion area. Data are expressed as mean ± SD. *p < 0.05, **p < 0.01.

Oil Red O staining showed that compared with the control group, large areas of continuous flaky red atheromatous plaques could be seen, which was mainly concentrated in the aortic arch and the abdominal aorta and the plaque area of aorta intima was significantly increased in model group (*p* < 0.01). Compared with model group, only scattered red atherosclerotic plaques could be seen and the plaque area of aorta intima was significantly decreased in simvastatin group (*p* < 0.01); a small amount of red atherosclerotic plaques was observed and the plaque area of aorta intima was significantly decreased in SMYA group (*p* < 0.05) (Figure 1).

**FIGURE 2:**
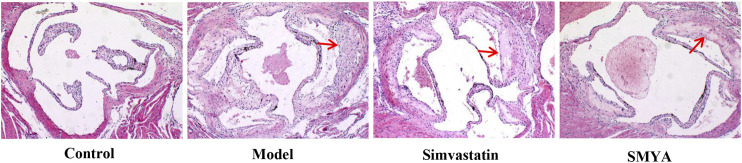
Effect of SMYA on Plaque of Aortic Root. HE staining showed: compared with control group, the plaque area of aortic root was increased significantly and there was no significant change of aorta lumen area in model group; the ratio of plaque to lumen area was significantly increased; plaques had the characteristics of thin fibrous cap and large lipid core and the area of aorta media was significantly decreased in model group. Compared with the model group, the plaque area of aortic root was significantly reduced in both the simvastatin and SMYA groups; there was no significant change in aorta lumen area in the two treatment groups; the ratio of plaque to lumen area was significantly decreased in the two treatment groups; the minimum fiber cap thickness was significantly increased, and there was no obvious rupture and hemorrhage of plaque; however, there was no significant change in the area of aorta media. Compared with the simvastatin group, the minimum fiber cap thickness was increased significantly in SMYA group. Red arrows marked the plaque area (Magnification, ×100).

**FIGURE 3:**
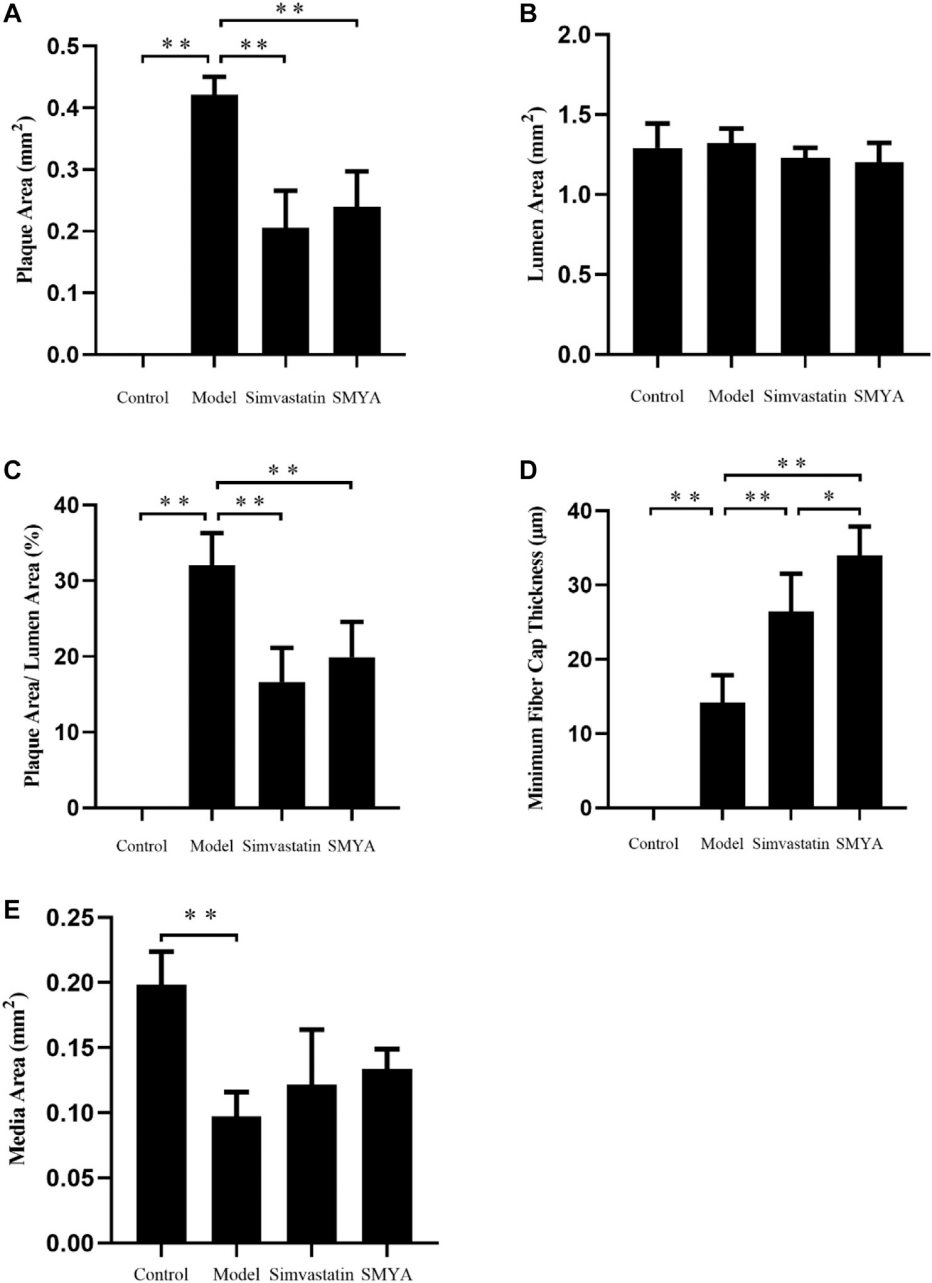
Quantitative analysis of SMYA on plaque of aortic root. **(A)** plaque area each group; **(B)** lumen area each group; **(C)** plaque area/lumen area each group; **(D)** minimum fiber cap thickness each group; **(E)** media area each group. Data are expressed as mean ± SD. *p < 0.05, **p < 0.01.

HE staining showed that compared with the control group, the plaque area of aortic root was increased significantly in model group (*p* < 0.01); there was no significant change in aorta lumen area (*p* > 0.05); the ratio of plaque to lumen area was significantly increased (*p* < 0.01); plaques had the characteristics of thin fibrous cap and large lipid core; a large number of foam cells and cholesterol crystal deposition could be seen in the plaques, and even plaques rupture and hemorrhage occurred; the aorta intima was significantly thickened, the smooth muscle of aorta media was disordered and atrophic, and the area of aorta media was significantly decreased (*p* < 0.01). Compared with the model group, the plaque area of aortic root was significantly reduced in both the simvastatin and SMYA groups (*p* < 0.01); there was no significant change in aorta lumen area (*p* > 0.05) in the two treatment groups; the ratio of plaque to lumen area was significantly decreased (*p* < 0.01) in the two treatment groups; the minimum fiber cap thickness was significantly increased (*p* < 0.01), and there was no obvious rupture and hemorrhage of plaque; however, there was no significant change in the area of aorta media (*p* > 0.05). Compared with the simvastatin group, the minimum fiber cap thickness was increased significantly in SMYA group (*p* < 0.05) (Figures 2 and 3).

### Effect of SMYA on VV Density

**FIGURE 4:**
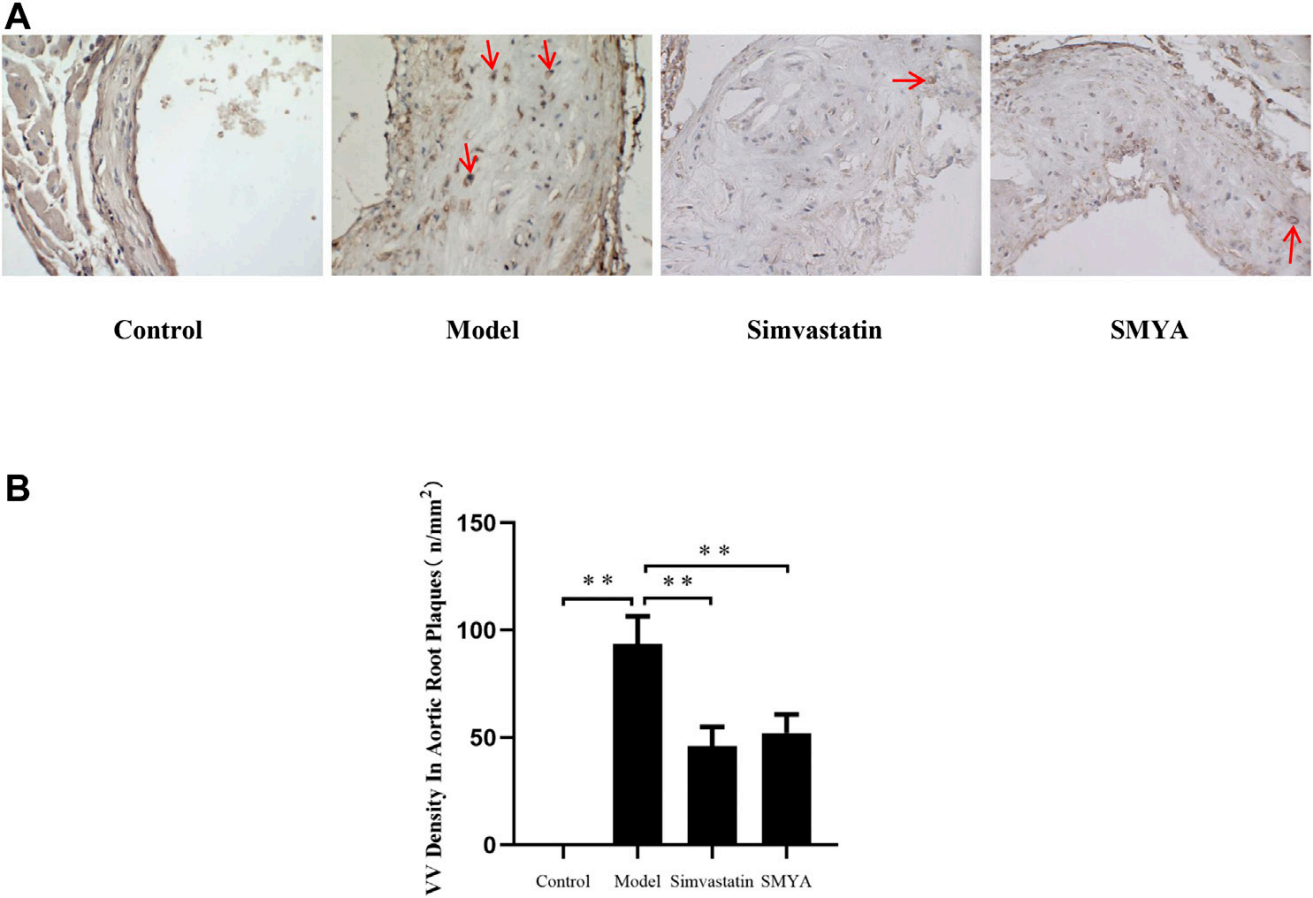
Effect of SMYA on VV density in aortic root plaques. **(A)** immunohistochemical staining showed: compared with the control group, the density of CD34 positive stained VV in aortic root plaques was significantly increased in model group. Compared with the model group, the VV density in aortic root plaques was significantly decreased in both the simvastatin and SMYA groups. Red arrows marked the VV neovascularization in plaque (Magnification, ×400). **(B)** quantitative analysis of SMYA on VV density in aortic root plaques. Data are expressed as mean ± SD. **p < 0.01.

Immunohistochemical staining showed that compared with the control group, the density of CD34 positive stained VV in aortic root plaques was significantly increased in model group (*p* < 0.01). Compared with the model group, the VV density in aortic root plaques was significantly decreased in both the simvastatin and SMYA groups (*p* < 0.01) (Figure 4).

**FIGURE 5:**
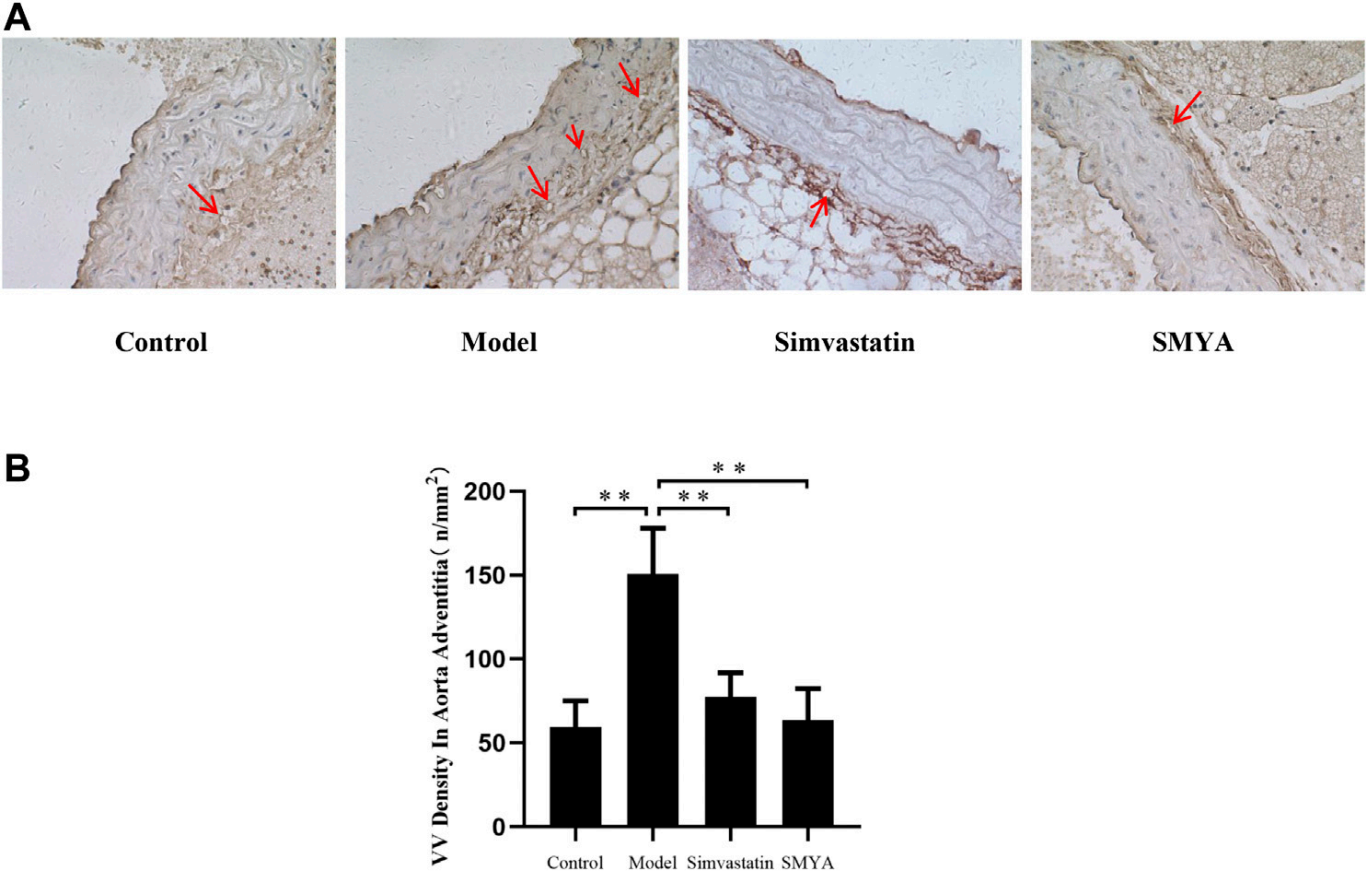
Effect of SMYA on VV density in aorta adventitia. Immunohistochemical staining showed: compared with the control group, the density of CD34 positive stained VV in aorta adventitia was significantly increased in model group. Compared with the model group, the VV density in aorta adventitia was significantly decreased in both the simvastatin and SMYA groups. Red arrows marked the VV in aorta adventitia (Magnification, ×400). **(B)** quantitative analysis of SMYA on VV density in aorta adventitia. Data are expressed as mean ± SD. **p < 0.01.

Compared with the control group, the density of CD34 positive stained VV in aorta adventitia was significantly increased in model group (*p* < 0.01). Compared with the model group, the VV density in aorta adventitia was significantly decreased in both the simvastatin and SMYA groups (*p* < 0.01) (Figure 5).

### Effect of SMYA on VV Maturation

**FIGURE 6:**
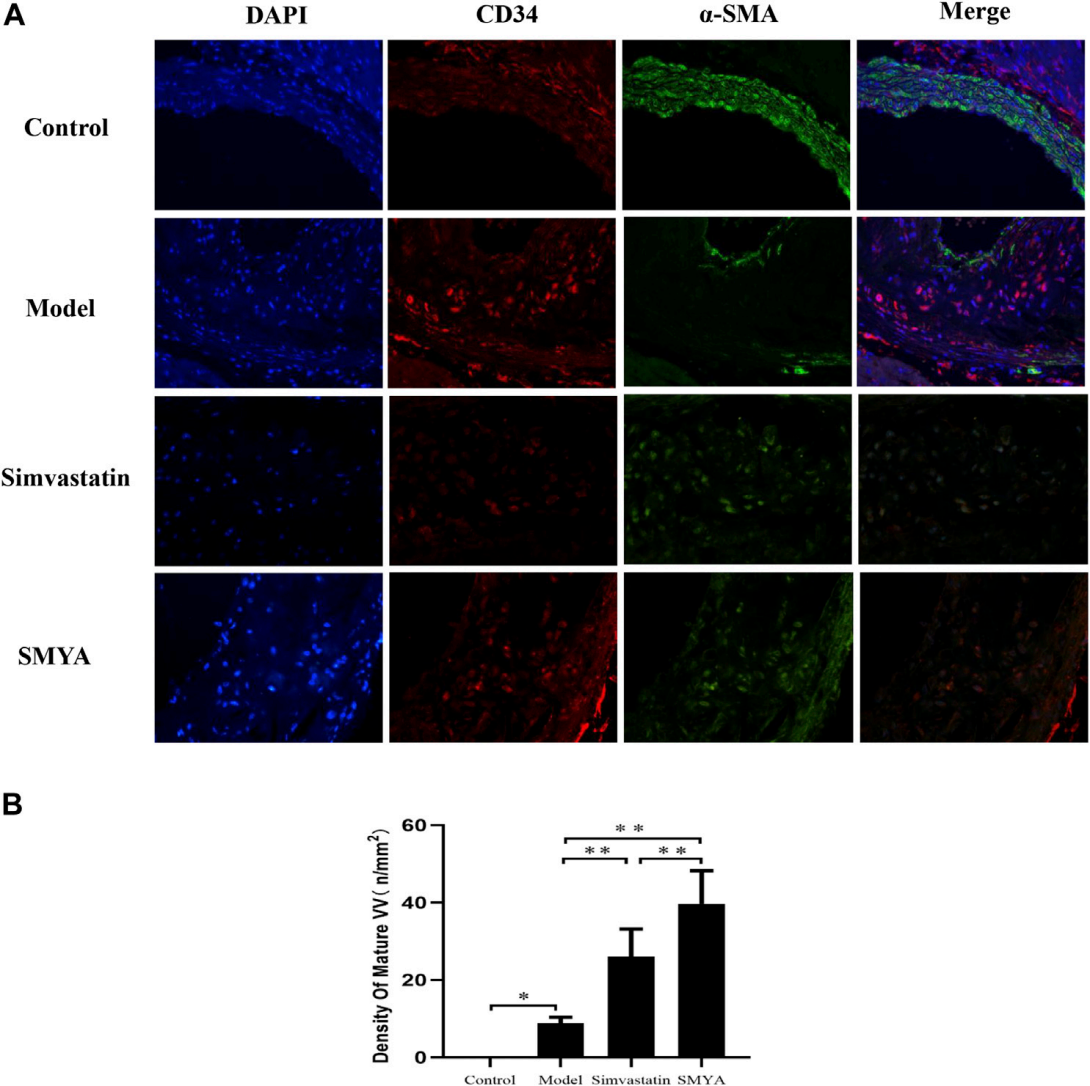
Effect of SMYA on VV Maturation. Double-labelling Immunofluorescence showed: in control group, there was no plaque in aortic root. In model group, there were a few mature VV covered by smooth muscle cells in aortic root plaques. Compared with the model group, the density of mature VV was significantly increased in aortic root plaques in both the simvastatin and SMYA groups. Compared with simvastatin group, the density of mature VV increased more significantly in SMYA group (Magnification, ×400). **(B)** quantitative analysis of SMYA on VV maturation in aortic root plaques. Data are expressed as mean ± SD. *p < 0.05, **p < 0.01.

The results of double-labelling immunofluorescence showed that in control group, there was no plaque formation in aortic root. In model group, there was fewer mature VV covered by smooth muscle cells in aortic root plaques. Compared with the model group, the density of mature VV in plaques was significantly increased in aortic root plaques in both the simvastatin and SMYA groups (*p* < 0.01). Compared with simvastatin group, the density of mature VV increased more significantly in SMYA group (*p* < 0.01). The results indicated that SMYA effectively promoted the recruitment of smooth muscle cells, promoted the maturation of new VV in plaques, and played a role in stabilizing vulnerable plaques, and its effect was greater than simvastatin (Figure 6).

### Effect of SMYA on HIF-1α-Apelin/APJ and Ang-1/Tie Signal Pathways

**FIGURE 7:**
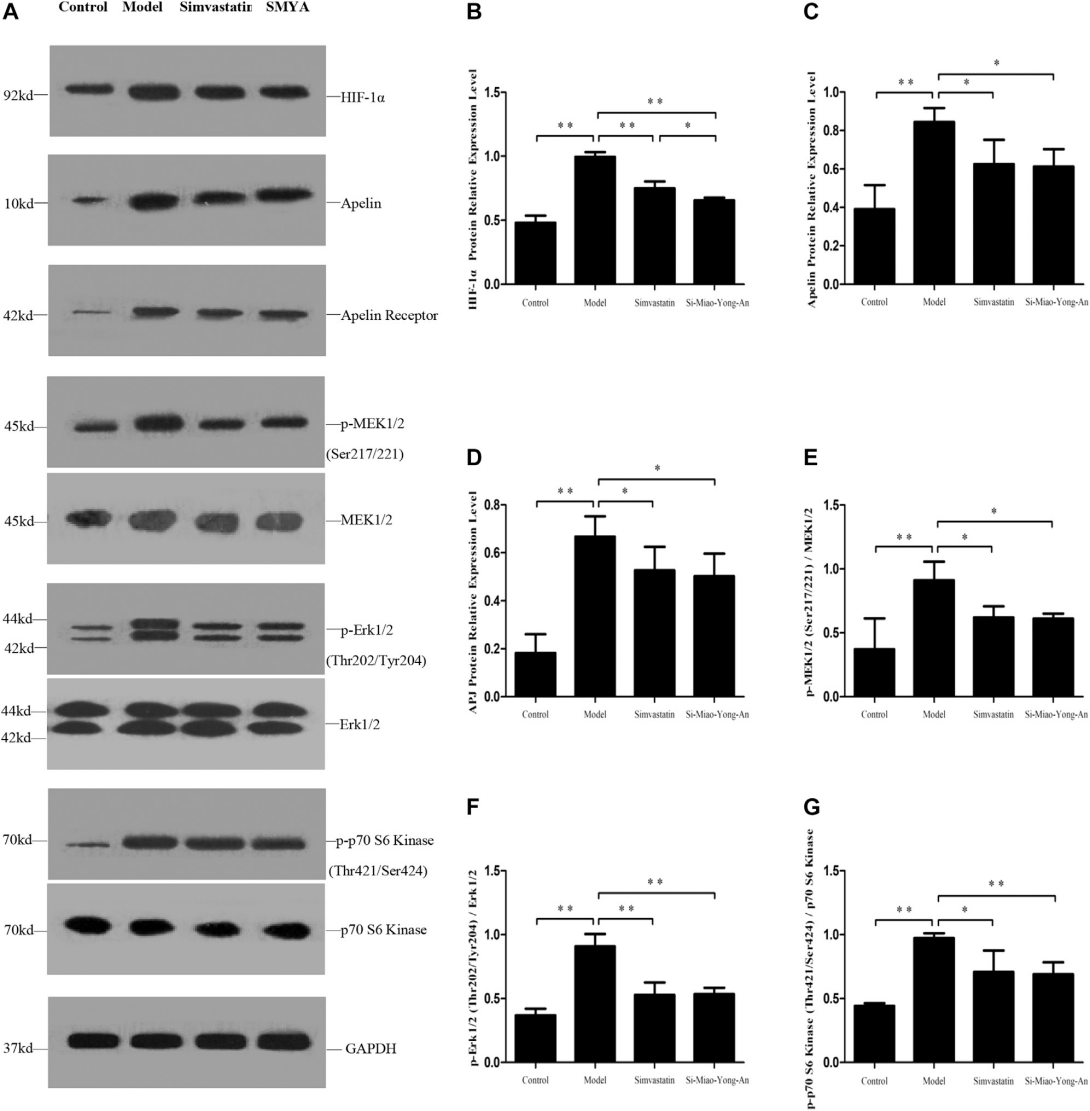
Effect of SMYA on HIF-1α-Apelin/APJ Signal Pathway. **(A)** Western blot was applied to detect the key proteins of HIF-1α-Apelin/APJ signal pathway in aorta of each group. B–G: Semi-quantitative analysis of the results. *p < 0.05, **p < 0.01.

Compared with the control group, the HIF-1α, Apelin-13, APJ, phospho-MEK1/2 (Ser217/221), phospho-Erk1/2 (Thr202/Tyr204) and phospho-p70 S6 Kinase (Thr421/Ser424) proteins expression increased in the model group (*p* < 0.01). Compared with the model group, the HIF-1α, Apelin-13, APJ, phospho-MEK1/2 (Ser217/221), phospho-Erk1/2 (Thr202/Tyr204) and phospho-p70 S6 Kinase (Thr421/Ser424) expression decreased in both of the SMYA and simvastatin groups (*p* < 0.01 or *p* < 0.05). Additionally, compared with the simvastatin group, the HIF-1α protein expression decreased more significantly in SMYA group (*p* < 0.05). This result indicated that SMYA effectively inhibited the HIF-1α-Apelin/APJ signal pathway (Figure 7).

**FIGURE 8:**
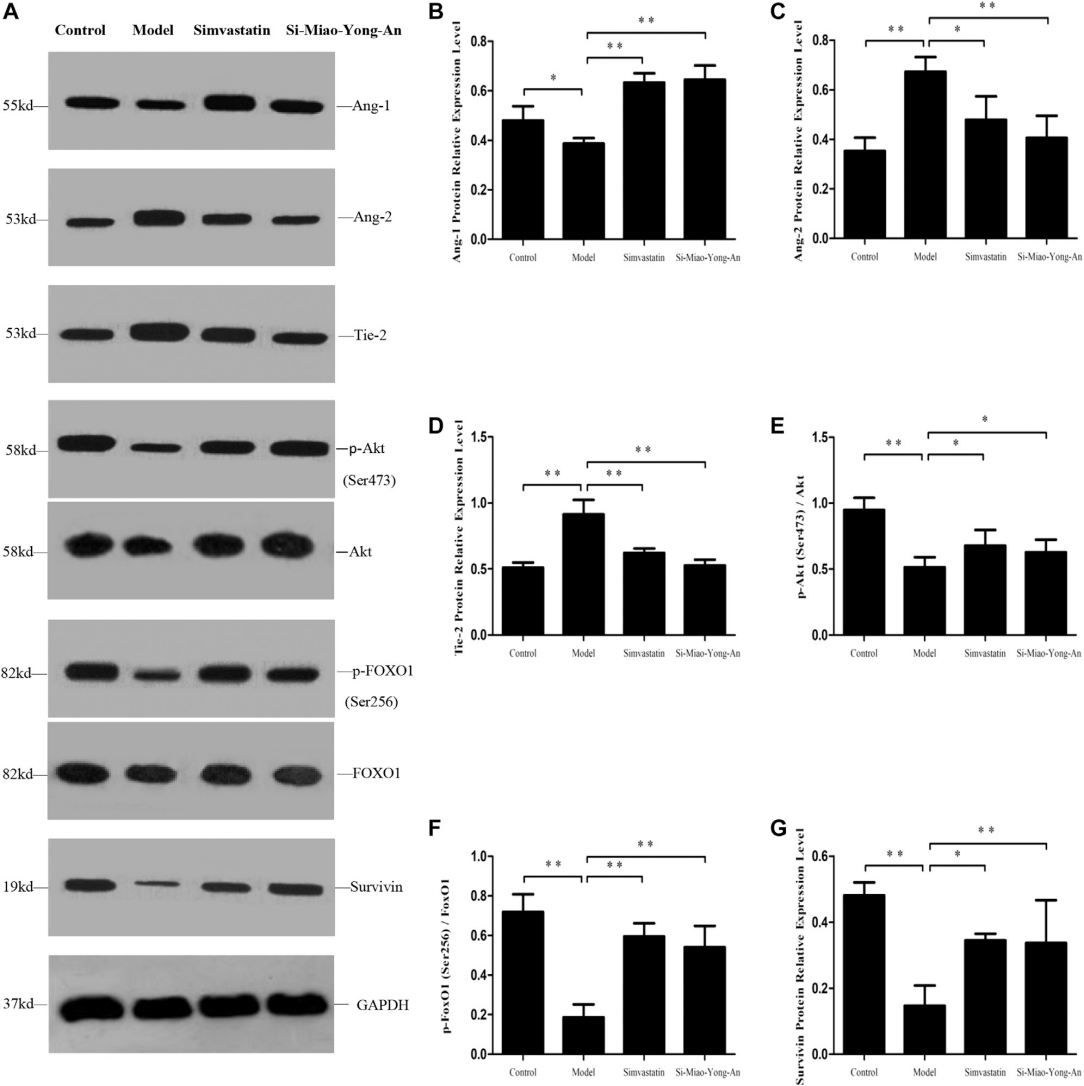
Effect of SMYA on Ang-1/Tie Signal Pathway. **(A)** Western blot was applied to detect the key proteins of Ang-1/Tie signal pathway in aorta of each group. B–G: Semi-quantitative analysis of the results. *p < 0.05, **p < 0.01.

Compared with the control group, the Ang-1, phospho-Akt (Ser473), phospho-FoxO1 (Ser256) and Survivin proteins expression decreased in model group (*p* < 0.01 or *p* < 0.05), however the Ang-2 and Tie-2 proteins were up-regulated (*p* < 0.01). Compared with the model group, the Ang-1, phospho-Akt (Ser473), phospho-FoxO1 (Ser256) and Survivin proteins increased in both of the SMYA and simvastatin groups (*p* < 0.01 or *p* < 0.05), and the Ang-2 and Tie-2 decreased significantly in both of the two treatment groups (*p* < 0.01 or *p* < 0.05). This result indicated that SMYA activated the Ang-1/Tie signal pathway (Figure 8).

## Discussion

In the early stage of AS, multiple active factors, such as reactive oxygen species, chemokines and inflammatory mediators, accumulate in the vascular adventitia and enter into the VV by microcirculation. These active factors adhere to the VV network and damage the transport function of VV. The hypoxia condition induces the HIF-1α expression (Wong et al., 2017; Jain et al., 2018). HIF-1α referred to as “master switch for hypoxia gene expression” is a DNA-binding transcription factor closely associated with specific nuclear cofactors (Fong, 2015; Ferns and Heikal, 2017; Yao et al., 2017; Heriansyah et al., 2018).

**FIGURE 9:**
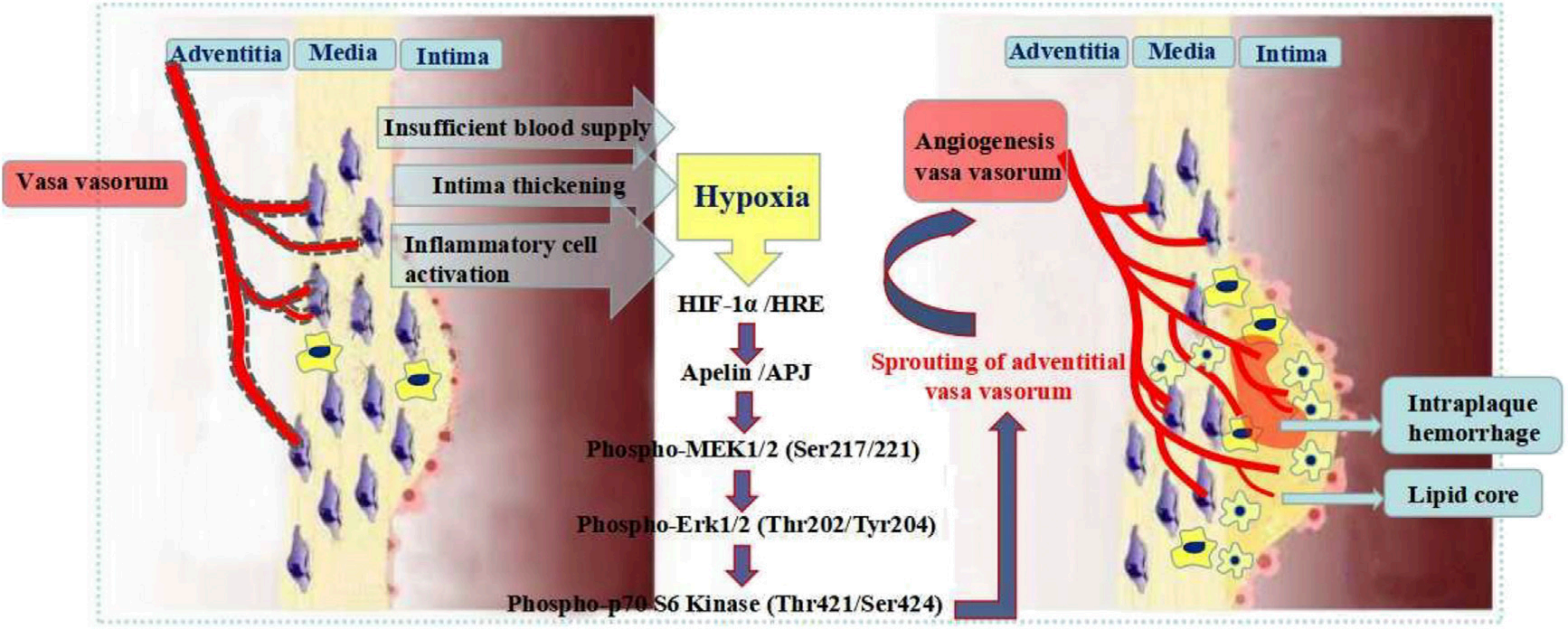
Mechanism of HIF-1α-Apelin/APJ signaling pathway regulating vasa vasorum angiogenesis.

Apelin is a target gene of HIF-1α. HIF-1α binds to the first intron of Apelin and triggers Apelin to release continuously. Apelin binds to the endogenous receptor APJ and promotes the endothelial cells (ECs) activation and proliferation (Eyries et al., 2008; Zimna and Kurpisz, 2015). The Apelin-APJ system plays an important role in embryonic vascular development and angiogenesis in adulthood (Wu et al., 2017; Cheng et al., 2019). Apelin-APJ actives the MEK/ERK signaling pathway and afterward actives p70 S6 kinase, which promotes VV angiogenesis (Yang et al., 2009; He et al., 2015). HIF-1α-Apelin/APJ signaling pathway plays a key role in VV angiogenesis (Figure 9). The study on the key proteins expression of the signaling pathway is of great significance for exploring the VV angiogenesis pathogenesis and the effective treatment of AS vulnerable plaques.

**FIGURE 10:**
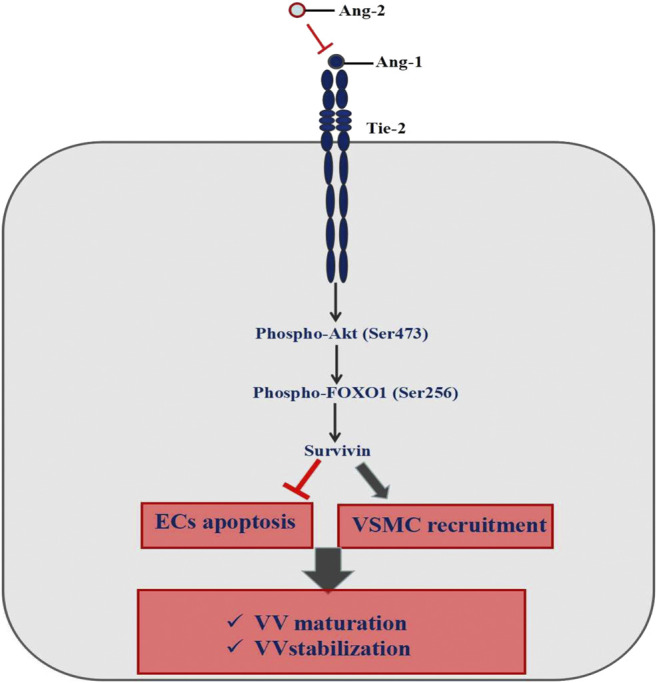
Mechanism of Ang-1/Tie signaling pathway maintaining the vasa vasorum maturation.

Immature VV must go through the following steps to form a mature vascular structure, including basement membrane formation—ECs recruiting parietal cells (pericytes and smooth muscle cells) to form mature blood vessels; branching, remodeling, and pruning of the vascular network to adapt to the local tissue requirements; arteriovenous differentiation (Sun et al., 2015). Ang1-Tie2 is an important pathway to maintain the stability of newly formed VV (Kim et al., 2000; Papapetropoulos et al., 2000; González-González et al., 2018; Akwii et al., 2019). Ang1 binding with Tie2 can activate the downstream PI3K/Akt pathway, then regulate FOXO1 protein phosphorylation on the serine site and make FOXO1 protein output from the nucleus, and inhibit DNA binding. It leads to a change in FOXO1 transcription activity, inhibits FOXO1 protein function and then promotes the Survivin protein expression, which eventually inhibits ECs apoptosis, promotes the parietal cells recruitment to form mature VV, and maintain the stability of VV (Kontos et al., 2002; Daly et al., 2004; Ohashi et al., 2004) (Figure 10).

Based on the above understanding, our study explored whether SMYA had effect on decreasing and stabilizing AS plaque by regulating VV remodeling. This research showed SMYA could significantly improve the pathological morphology of aortic plaques in mice, reduce plaque area and plaque to lumen area ratio, and increase minimum fibrous cap thickness to stabilize plaque. The effect of SMYA on the fiber cap thickness was obviously superior to that of simvastatin. SMYA could effectively inhibit VV formation to stabilize plaque. SMYA could promote the recruitment of smooth muscle cells, which made the new VV surrounded by smooth muscle cells and promoted the new VV maturation in plaque. The mechanism study showed that SMYA could inhibit VV angiogenesis by restraining HIF-1α-Apelin/APJ signal pathway. SMYA could promote VV maturation by activating Ang-1/Tie signal pathways.

However, this study only discussed the effect and mechanism of SMYA on the AS plaques at one point-in-time, and did not explain the differences and changes of the pathological mechanism in different stages of AS formation and the differences of the intervention effect and mechanism of SMYA.

## Conclusion

SMYA can inhibit VV angiogenesis by regulating the HIF-1α-Apelin/APJ signaling pathway; can promote the newborn VV maturation by regulating the Ang-1/Tie signaling pathway, thus stabilizing AS vulnerable plaque.

## Data Availability

The original contributions presented in the study are included in the article/Supplementary Material, further inquiries can be directed to the corresponding author.
